# Rapid and Sensitive Detection of *Schistosoma mansoni* in the Intermediate Snail Hosts Using Loop-Mediated Isothermal Amplification (LAMP) Diagnostics

**DOI:** 10.3390/tropicalmed11060157

**Published:** 2026-06-11

**Authors:** Hong-Mei Li, Zhi-Qiang Qin, Shan Lv, Jing Xu, Nicholas Midzi, Masceline Jenipher Mutsaka-Makuvaza, Ting Feng, Robert Bergquist, Xiao-Nong Zhou

**Affiliations:** 1 National Institute of Parasitic Diseases, Chinese Center for Disease Control and Prevention (Chinese Center for Tropical Diseases Research), NHC Key Laboratory of Parasite and Vector Biology, WHO Collaborating Centre for Tropical Diseases, National Center for International Research on Tropical Diseases, Shanghai 200025, China; lihm@nipd.chinacdc.cn (H.-M.L.); qinzq@nipd.chinacdc.cn (Z.-Q.Q.); lvshan@nipd.chinacdc.cn (S.L.); xujing@nipd.chinacdc.cn (J.X.); fengting@nipd.chinacdc.cn (T.F.); 2 National Institute of Health Research, Ministry of Health and Child Care, Harare P.O. Box CY 573, Zimbabwe; nmidzi@mohcc.org.zw (N.M.); mascelinejeni@gmail.com (M.J.M.-M.); 3 Department of Microbiology and Parasitology, School of Medicine and Pharmacy, College of Medicine and Health Sciences, University of Rwanda, Butare P.O. Box 117, Rwanda; 4 Geospatial Health, Ingerod, SE-454 94 Brastad, Sweden; robert.bergquist@outlook.com

**Keywords:** *Schistosoma mansoni*, LAMP, ND1, snail surveillance, molecular diagnosis, schistosomiasis

## Abstract

Schistosomiasis is an important snail-borne neglected tropical disease, and detecting infected snails is a priority for its control and elimination. However, conventional parasitological methods, such as crushed snails and cercarial shedding, have limited sensitivity. In this study, we developed a novel loop-mediated isothermal amplification (LAMP) assay (smND1-LAMP) targeting the mitochondrial NADH dehydrogenase subunit 1 (ND1) gene of *Schistosoma mansoni*. The assay was optimized at 65 °C for 1 h and demonstrated a detection limit of one copy of the pUC57/smND1 recombinant plasmid. Its diagnostic performance was evaluated using laboratory-infected *Biomphalaria* snails and field-collected samples from Zimbabwe and Burkina Faso, and compared with microscopy, conventional PCR and SYBR Green real-time PCR (SGPCR). In laboratory experiments, smND1-LAMP achieved 100% specificity and 75% sensitivity, outperforming microscopy and showing a similar performance to SGPCR. In field surveys, smND1-LAMP detected a higher positive rate (25.9%) than conventional PCR (22.2%) in Burkina Faso, while microscopy failed to identify any positive snails. Both molecular methods identified infections that were missed by parasitological techniques. These findings demonstrate that smND1-LAMP assay is a sensitive, specific, and field-applicable tool for detecting *S. mansoni* infection in snails. It provides an effective alternative for routine surveillance and early warning of changing schistosomiasis endemicity.

## 1. Introduction

Schistosomiasis is a parasitic disease caused by blood flukes (*Schistosoma* spp.), whose definitive stages reside in the human intestinal or urogenital systems, depending on the species. Its life cycle involves intermediate snail hosts that release the infectious larval stages, the cercariae, which are capable of penetrating human skin [1]. Within snails, schistosomes undergo multiple developmental stages, including miracidia, mother sporocysts, daughter sporocysts and cercariae, with substantial morphological changes. While cercariae can be readily identified microscopically with their distinct morphological features, earlier developmental stages, such as miracidia and sporocysts, are difficult to distinguish under the microscope. In addition, co-infection with other animal parasites may further complicate the identification of early-stage infections based solely on morphology [2].

Classified by the World Health Organization (WHO) as a priority neglected tropical disease (NTD) [3], schistosomiasis is a disease caused by several species that infect humans, including *S. mansoni*, *S. haematobium*, *S. japonicum*, *S. mekongi*, *S. intercalatum*, and *S. guineensis*, with the majority of infections being due to the first three species [4,5]. Among these, *S. mansoni* causes intestinal schistosomiasis, which is endemic in 54 countries and regions, primarily in Africa and South America, but also in parts of the Middle East and the Caribbean [6,7]. In recent years, imported cases of schistosomiasis due to *S. mansoni* have been documented in non-endemic countries [8], raising concerns about the potential for local transmission [9]. For example, although *S. japonicum* is the only endemic species causing schistosomiasis in China [10], *S. mansoni* has attracted increasing attention since the expanding distribution of *Biomphalaria straminea*, one of the intermediate host snail species, has been reported in multiple areas of Shenzhen in southern China, attributed to accidental importation [11].

In the second roadmap for NTDs, WHO has set the ambitious goal of eliminating schistosomiasis as a public health problem by 2030 [12]. Achieving this goal relies heavily on the availability of accurate and highly sensitive diagnostic tools [13], the development of which for snail diagnosis have been highlighted as a key priority in the WHO guidelines for schistosomiasis control and elimination programmes [14].

The Kato–Katz technique remains the standard parasitological method for diagnosing *S. mansoni* infections by detecting eggs of the parasite in feces [15]. Finding the infection in the intermediate host snail generally rests on parasitological methods, such as cercarial shedding and snail crushing, which rely on the observation of cercariae emerging from infected snails [16]. Although these methods are simple and cost-effective, the low sensitivity leading to false negative results limits their application in areas with low infection prevalence and those undergoing preventive chemotherapy programmes [13]. Immunological methods offer higher sensitivity but may suffer from lower specificity compared to parasitological techniques [17], whereas molecular diagnostic methods provide both high sensitivity and specificity, leading to their increasing use [18].

Molecular detection relies on the amplification of parasite DNA using species-specific target sequences, with performance being largely dependent on the specificity of the selected target. Some assays target conserved genomic regions for pan-*Schistosoma* detection, which is advantageous for screening in settings characterized by the presence of two or more different schistosome species [19]. Conventional and real-time polymerase chain reaction (PCR) require thermal cycling equipment [20]. By contrast, loop-mediated isothermal amplification (LAMP) and recombinant polymerase amplification (RPA)-based assays are isothermal amplification techniques that may not require sophisticated instrumentation, potentially offering cost-effectiveness and user-friendly field application [21,22,23]. LAMP employs four specific primers that target six distinct regions of the target DNA and utilizes a strand displacement mechanism for rapid amplification under isothermal conditions [24]. Amplification can be detected in real time via visual colour change or using simple instruments.

LAMP technology has been successfully applied in order to detect schistosome infections in snails [18] and has played an important role in surveillance during the schistosomiasis elimination phase in China [25,26]. However, previous studies have shown that the diagnostic performance of LAMP-based assays varies depending on the selected genetic target, highlighting the need for further methodological optimization [27]. LAMP is considered to be a promising tool worth further investigation as the currently limited number of relevant studies prevents a definitive conclusion regarding its large-scale applicability [28]. This study integrated bioinformatics analysis with nucleic acid detection technology to identify an optimal LAMP-based molecular approach for *S. mansoni*. The developed assay was then applied to detect *S. mansoni* infections in *Biomphalaria* snails, with the aim of improving intermediate host surveillance and supporting more effective disease control.

## 2. Materials and Methods

### 2.1. Sample Sources

The samples used in this study included parasite materials representing different species and life cycle stages, laboratory-maintained snails, and field-collected snails. With regard to parasite species, we used cercariae and adult *S. mansoni*, adult *S. japonicum*, *S. haematobium* eggs, adult *Clonorchis sinensis*, adult *Paragonimus westermani* and adult *Echinococcus granulosus*. These parasites are available at the National Institute of Parasitic Diseases (NIPD), Chinese Center for Disease Control and Prevention (China CDC), Shanghai, China. Detailed information of the different parasite sources is provided in Table S1.

For laboratory infection experiments, the intermediate snail host *Bi. glabrata* is maintained and propagated at NIPD. For field validation, *Bi. pfeifferi* and *Bulinus globosus* snails were collected in Zimbabwe and Burkina Faso during field surveys. Field-collected snails were preserved in ethanol samples and rinsed three times with distilled water before DNA extraction to reduce residual ethanol carry-over. DNA was extracted using the Qiagen DNeasy Blood & Tissue kit (Qiagen, Hilden, Germany) according to the manufacturer’s instructions, as described below.

### 2.2. Bioinformatic Selection of Target Regions and LAMP Primers Design

Mitochondrial gene sequences of *S. mansoni* (GenBank accession: NC_002545), *S. haematobium* (GenBank accession: NC_008074), *S. japonicum* (GenBank accession: NC_002544) and *S. mekongi* (GenBank accession: NC_002529) were retrieved from GenBank and aligned using DNAMAN software (version 9.0, Lynnon Biosoft, San Ramon, CA, USA, https://www.lynnon.com, accessed on 2 January 2020) to identify candidate regions with genetic conservation and high interspecies divergence. Pair-wise comparisons between *S. mansoni* and three representative *Schistosoma* species, namely *S. haematobium*, *S. japonicum* and *S. mekongi*, showed homology values ranging from 60.2% to 82.1% across the candidate mitochondrial regions (Table S2).

Based on sequence homology, five mitochondrial target regions of *S. mansoni* were selected for LAMP primer screening: s-rRNA, ND1, CYTB, ND4 and COX3 (Table S3).

LAMP primer sets targeting the five candidate regions were designed using Primer explorer V5 (Eiken Chemical Co., Ltd., Tokyo, Japan, https://primerexplorer.eiken.co.jp/e/, accessed on 27 August 2020). Each primer set consisted of four primers: a forward outer primer (F3), forward inner primer (FIP), backward inner primer (BIP) and backward outer primer (B3). The final primer sequences were provided in Table 1.

### 2.3. LAMP Reaction System and Results Interpretation

Using *S. mansoni* DNA as the positive control template, distilled water as the negative control template and DNA from test samples as the experimental templates, the LAMP assay was performed using a reaction system adapted from our previously established protocol for *S. japonicum* [21], with modifications for the present *S. mansoni*-specific assay. The modifications included replacement with smND1-specific primers, optimization of the reaction conditions, and refinement of the interpretation methods. The final concentrations of various reagents in a 25 µL LAMP reaction system were as follows: 20 mM Tris-HCl (pH 8.8); 150 mM KCl; 10 mM (NH_4_)_2_SO_4_; 8 mM MgSO_4_; 0.1% Tween-20; 1.4 mM dNTP mixture; 0.8 M betaine; 0.05 µM F3 and B3 primers; 0.4 µM FIP and BIPs; 8 U Bst polymerase; and 1 µL DNA template (approximately 1–10 ng). The reaction volume was adjusted to 25 µL with distilled water. The reaction was incubated at 60–65 °C for 1 h followed by enzyme inactivation at 80 °C for 10 min, and the optimal reaction temperature requires experimental identification.

LAMP results were interpreted using three methods. (1) First, visual observation, in which 1 μL calcein dye (50 µM Calcein and 1 mM MnCl_2_) was added to the reaction mixture before amplification to allow closed-tube visual detection and reduce post-amplification contamination risk. Reactions showing green fluorescence were regarded as positive, whereas reactions remaining yellow/orange were regarded as negative. (2) Second, agarose gel electrophoresis, in which amplification products were separated on a 2% agarose gel and visualized under UV light, where the presence of the characteristic LAMP bands was interpreted as positive and their absence as negative. (3) Third, turbidity measurement, where reaction tubes were placed in a LAMP turbidimeter and the real-time turbidity changes caused by magnesium pyrophosphate precipitation were monitored, with turbidity values > 0.1 regarded as positive and ≤0.1 as negative.

### 2.4. Primer Screening, Validation, and Optimization

To identify the optimal LAMP primer set for *S. mansoni* detection, five primer sets (Section 2.2) were screened using DNA from adult *S. mansoni*, *S. mansoni* cercariae, adult *S. japonicum*, and distilled water as a negative control. Reactions were conducted at 65 °C for 1 h and initially evaluated by a visual colour change. The optimal primer set was assessed based on the following criteria: (i) no colour change in the negative control; (ii) no cross-reactivity with non-target DNA; and (iii) positive amplification exclusively from *S. mansoni* DNA. Among these, primer sets yielding faster and stronger colour development were considered superior.

To validate the selected target region, conventional PCR was performed using the F3 and B3 primers from the optimal LAMP primer set and *S. mansoni* cercarial DNA as the template. PCR was performed in a total volume of 20 µL, containing 1.25 U of Taq DNA polymerase, 2 µL of 10 X Taq Buffer [Tris-HCl (pH 8.9) 100 mM, KCl 500 mM, MgCl_2_ 15 mM], 0.2 mM dNTP Mixture, 1 µL of each primer (10 µM each), and 1 µL of DNA template (approximately 1–10 ng), and nuclease-free water to 20 µL. The cycling conditions were as follows: initial denaturation at 94 °C for 3 min; 35 cycles of denaturation at 94 °C for 30 s; annealing at 56 °C for 45 s; and extension at 72 °C for 50 s, followed by a final extension at 72 °C for 7 min. PCR products were analyzed by 2% agarose gel electrophoresis to confirm the expected fragment size. The same template was then tested by LAMP using the selected LAMP primer set and evaluated by the three methods described in Section 2.3.

Reaction temperatures of 65 °C, 63 °C and 60 °C were compared to determine the optimal amplification condition based on amplification onset time, reaction signal intensity and negative control performance. The best-performing primer set and reaction temperature were selected as the optimized LAMP conditions.

Specificity was evaluated using DNA templates from *S. mansoni* adults and cercariae, *S. japonicum* adults, *S. haematobium* eggs, *E. granulosus* adults, *P. westermani* adults and *C. sinensis* adults. LAMP reactions were conducted under the optimized conditions described above, and nuclease-free water was included as a no-template control in each run. For sensitivity testing, the full-length smND1 fragment was synthesized and cloned into the pUC57 vector to generate a recombinant plasmid, which was confirmed by sequencing and used as a positive control plasmid. The plasmid concentration was measured and the copy number calculated before serial dilution. LAMP reactions were then performed using 10-fold serial dilutions of the recombinant plasmid ranging from 10^7^ to 10^−1^ copies per reaction (10^7^, 10^6^, 10^5^, 10^4^, 10^3^, 10^2^, 10, 1 and 10^−1^ copies) to determine the detection limit. All experiments were performed in triplicate. The detection limit was defined as the lowest plasmid copy number that produced a reproducible positive result under the testing conditions.

### 2.5. Evaluation Using Laboratory-Infected Bi. Snails

To evaluate diagnostic performance under laboratory conditions, *Bi. glabrata* snails exposed to *S. mansoni* miracidia for 61 days, along with uninfected snails of the same species, were tested using parasitological and molecular methods. The snails were first examined for infection by cercarial shedding and snail crushing. For cercarial shedding, individual snails were placed in dechlorinated water and exposed to light for 4 h, after which the water was examined under a dissecting microscope for released cercariae. For snail crushing, each snail was gently squashed under a glass plate, shell fragments were removed, and the soft tissue was examined microscopically for cercariae [16,29]. After microscopic examination, each snail was processed individually. Soft tissue was preserved, and DNA was extracted from the tissue of each individual snail under study as described above. The extracted DNA was used as the template for both the optimized LAMP assay and SYBR Green real-time PCR (SGPCR) to detect *S. mansoni* infection. SGPCR, a technique that quantifies DNA amplification with a fluorescent dye, was performed using the F3 and B3 primers from the optimal LAMP primer set. Each SGPCR reaction was performed in a final volume of 20 µL, including 10 µL of SYBR Green Real time PCR Master mix (TOYOBO, Osaka, Japan), 0.4 µM of each primer, 1 µL of DNA template (approximately 1–10 ng) and nuclease-free water to 20 µL. The amplification conditions were as follows: initial denaturation at 95 °C for 3 min, followed by 40 cycles of denaturation at 95 °C for 15 s, annealing at 56 °C for 15 s, extension at 72 °C for 45 s and a final extension of 10 min at 72 °C.

### 2.6. Evaluation Using Field-Collected Bi. Snails

The optimized LAMP assay was further applied to detect *S. mansoni* infection in field-collected *Bi.* snails from schistosomiasis-endemic areas in Chevakadzi Ward, Zimbabwe, collected in 2023 [30] and from Lioulgou and Panamasso villages in Burkina Faso, collected in 2019, respectively. Snail specimens were first identified morphologically according to shell characteristics and infection was preliminarily assessed by cercarial shedding and snail crushing as described above. In Zimbabwe, field-collected *Bi. pfeifferi* snails were pooled by size, with approximately five snails per pool corresponding to about 400 mg of total snail tissue, to improve detection efficiency and save testing costs. To ensure that each pool could be linked with its collection site, only snails from the same sampling site were pooled together, and snails from different sites were not mixed. A total of 68 pooled *Bi. pfeifferi* samples and 5 pooled *Bu. globosus* samples were collected from five endemic villages. In Burkina Faso, 54 pooled *Bi. pfeifferi* snails were collected from two endemic villages. All samples were preserved in 500 μL of 96% ethanol. DNA extraction was performed as described for laboratory snails. Extracted DNA was used as the template for both optimized LAMP assay and conventional PCR. Selected LAMP- and PCR-positive samples were further confirmed by gene sequencing using the F3 and B3 primers from the optimal LAMP primer set.

### 2.7. Statistic Analysis

Snail exposure status (exposed vs. unexposed) was used as the reference standard to assess the diagnostic performance of the parasitological methods, the LAMP assay, and SGPCR, with experimentally exposed snails considered infected (positive) and unexposed snails considered uninfected (negative). Sensitivity and specificity were calculated as follows: sensitivity = TP/(TP + FN) × 100% and specificity = TN/(TN + FP) × 100%, where TP = true positives, TN = true negatives, FP = false positives, and FN = false negatives. The 95% confidence intervals (CIs) were calculated for sensitivity and specificity to reflect the precision of these estimates. The positive rate for each method was calculated as follows: positive rate = (number of test-positive samples/total number of samples) × 100%. Agreement between smND1-LAMP and the comparator methods, including microscopy, conventional PCR and SGPCR, was assessed using Cohen’s kappa coefficient. The kappa value was interpreted as follows: <0.20—poor agreement; 0.21—0.40—fair agreement; 0.41—0.60—moderate agreement; 0.61—0.80—substantial agreement; and 0.81—1.00—almost perfect agreement.

## 3. Results

### 3.1. Selection of the ND1 Target and Optimal LAMP Primer Set

**Figure 1:**
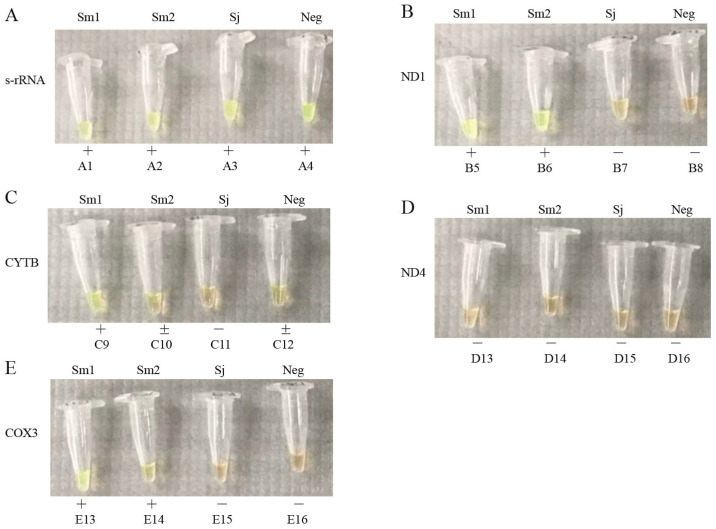
Screening results of five *S. mansoni* candidate LAMP primer sets. (**A**) s-rRNA primer set; (**B**) ND1 primer set; (**C**) CYTB primer set; (**D**) ND4 primer set a; (**E**) COX3 primer set. In all photos, Sm1 = adult *S. mansoni* DNA templates; Sm2 = *S. mansoni* cercariae DNA templates; Sj = adult *S. japonicum* DNA templates; Neg = negative control (distilled water). A green colour indicated a positive colour reaction, whereas yellow/orange indicated no colour reaction (negative). The result for each reaction was also labelled as positive (+), weak positive (±), or negative (−).

Five candidate mitochondrial targets were screened for LAMP assay development. The s-rRNA primer set produced non-specific amplification, including false positive reactions in negative controls, indicating poor specificity (Figure 1A). The ND4 primer set showed no specific amplification reaction (Figure 1D). The CYTB primer set specifically amplified *S. mansoni* DNA but showed weak background signals in the negative control (Figure 1C). In contrast, both the ND1 and COX3 primer sets showed good specificity, with amplification observed only in *S. mansoni* samples and no cross-reactivity with *S. japonicum* or the negative control (Figure 1B,E).

**Figure 2:**
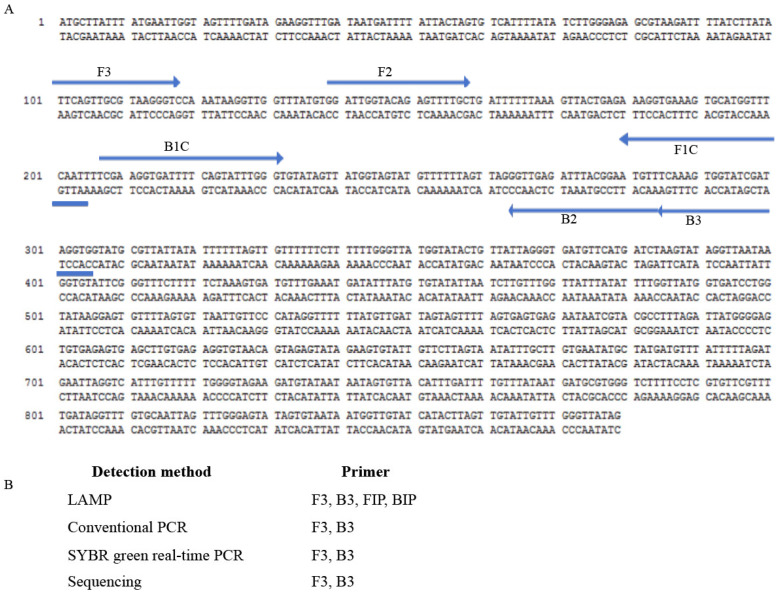
Schematic representation of the ND1-based LAMP primer design for *S. mansoni*. (**A**) LAMP primers designed based on the mitochondrial ND1 gene sequence of *S. mansoni*. Arrows indicate primer positions: F3 = forward outer primer; B3 = backward outer primer; FIP (F1C and F2 combined) = forward inner primer; and BIP (B1C and B2 combined) = backward inner primer. (**B**) Primer sets used in the four detection methods.

Based on cross-reactivity, amplification speed, and signal intensity, the primer set targeting a previously unexploited region of mitochondrial ND1 gene of *S. mansoni* (NADH dehydrogenase subunit 1; GenBank accession: NC_002545.1) was selected as the optimal target for assay development. The primer design is shown in Figure 2, and the primer sequences are provided in Table 1. The resulting assay was designated smND1-LAMP.

### 3.2. Validation of the smND1-LAMP Assay

**Figure 3:**
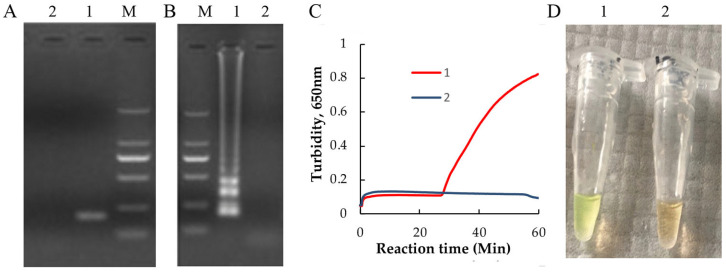
Validating of *S. mansoni* ND1 target by conventional PCR and smND1-LAMP. (**A**) Conventional PCR; (**B**) LAMP visualized by gel electrophoresis; (**C**) LAMP monitored by turbidity measurement; and (**D**) LAMP visualized by colour change. M: DL2000 DNA marker (bands from top to bottom: 2000 bp, 1000 bp, 750 bp, 500 bp, 250 bp and 100 bp); lane 1: *S. mansoni* cercarial DNA; lane 2: negative control (distilled water). The *S. mansoni* ND1 fragment is approximately 200 bp.

Using *S. mansoni* cercarial DNA as the template and the ND1-F3 and ND1-B3 primers, conventional PCR successfully amplified an approximately 200 base-pair (bp) fragment of the ND1 target gene (Figure 3A). The smND1-LAMP assay yielded concordant positive results, as confirmed by gel electrophoresis (Figure 3B), turbidity measurement (Figure 3C), and visual colour change (Figure 3D). The multiple bands observed in the LAMP gel electrophoresis represented the characteristic ladder-like amplification products generated during the LAMP reaction.

### 3.3. Optimization of the SmND1 LAMP Assay

**Figure 4:**
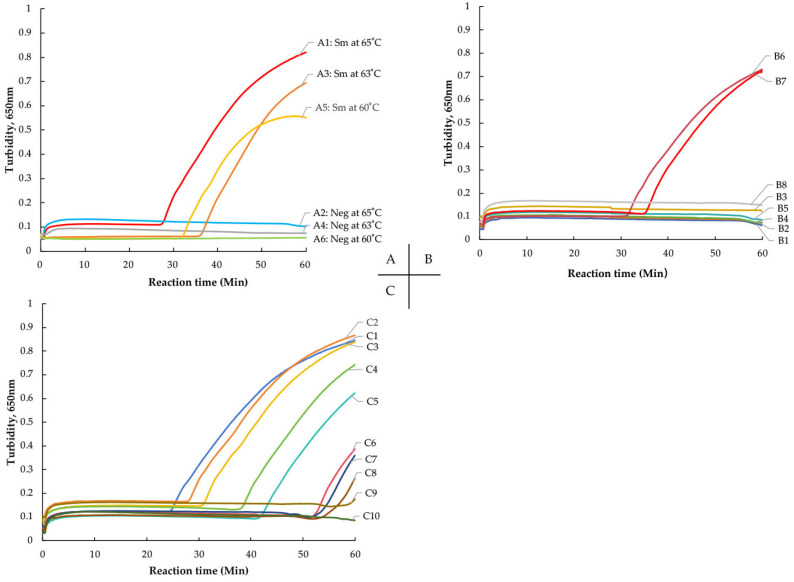
Optimization of the smND1-LAMP Assay. (**A**) Reaction temperature optimization. Lanes A1–A6 show amplification results at different temperatures for 1 h using *S. mansoni* DNA or negative control (distilled water) as template. Lane A1: *S. mansoni* DNA at 65 °C; Lane A2: negative control at 65 °C; Lane A3: *S. mansoni* DNA at 63 °C; Lane A4: negative control at 63 °C; Lane A5: *S. mansoni* DNA at 60 °C; Lane A6: negative control at 60 °C. (**B**) Specificity testing. Lane B1: *S. japonicum* DNA; Lane B2: *S. haematobium* DNA; Lane B3: *C. sinensis* DNA; Lane B4: *E. granulosus* DNA; Lane B5: *P. westermani* DNA; Lane B6: adult *S. mansoni* DNA; Lane B7: *S. mansoni* cercarial DNA; Lane B8: negative control. (**C**) Limit of detection. Lanes C1–C9: serial ten-fold dilutions (10^7^–10^−1^ copies) of the pUC57/smND1 (*S. mansoni*) recombinant positive plasmid; Lane C10: negative control (distilled water). Reactions shown in (**B**,**C**) were performed at 65 °C for 1 h.

As shown in Figure 4A, amplification was observed at all three tested temperatures. The amplification signal appeared at approximately 32 min at 60 °C, 37 min at 63 °C, and 29 min at 65 °C after initiation. No amplification was observed in the negative controls within 1 h at any tested temperature. Because 65 °C produced the earliest detectable signal and maintained negative control specificity, it was selected as the optimal reaction temperature for the smND1-LAMP assay.

### 3.4. Detection of Infection in Laboratory-Infected Bi. Snails

Under optimized conditions, the smND1-LAMP assay was conducted at 65 °C for 1 h, followed by enzyme inactivation at 80 °C for 10 min. Amplification results were interpreted using three methods: gel electrophoresis; turbidity measurement; and visual colour change. For visual detection, 1 μL of calcein dye was added to the 25 μL reaction system prior to amplification. These optimized conditions were applied in all subsequent experiments.

In the specificity evaluation, the smND1-LAMP assay specifically detected DNA from *S. mansoni* adults and cercariae, with no cross-reactivity observed with DNA from *S. haematobium*, *S. japonicum*, *E. granulosus*, *P. westermani* or *C. sinensis* (Figure 4B).

In sensitivity testing, amplification occurred earliest at higher recombinant plasmid copy number and was progressively delayed as the copy number decreased. No amplification was detected within 1 h at 0.1 copy, whereas positive amplification was observed at 1 copy. Therefore, the preliminary limit of detection of the smND1-LAMPassay was demonstrated to be one copy of the pUC57/smND1 recombinant plasmid per reaction (Figure 4C).

All six uninfected laboratory-maintained snails tested negative by both microscopy (cercarial shedding and snail crushing) and molecular methods (smND1-LAMP and SGPCR). Among the twelve snails experimentally exposed to *S. mansoni*, cercariae were observed in six, while the remaining six showed no cercarial shedding. The smND1-LAMP assay correctly identified all microscopy-positive snails, producing strong amplification signals. Among the six exposed but microscopy-negative snails, three were positive by smND1-LAMP, suggesting possible low-intensity infections. SGPCR yielded results identical to those of smND1-LAMP (Table 2). Microscopy achieved 100% specificity (6/6, 95% CI: 54.1–100%) but lower sensitivity (50%, 6/12, 95% CI: 21.1–78.9%), whereas both smND1-LAMP and SGPCR achieved 100% specificity (6/6, 95% CI: 54.1–100%) and higher sensitivity (75%, 9/12, 95% CI: 42.8–94.5%). Cohen’s kappa analysis showed substantial agreement (κ = 0.667) between smND1-LAMP and the microscopy-based methods, including cercarial shedding and snail crushing, and very high agreement (κ = 1.000) between smND1-LAMP and SGPCR.

### 3.5. Detection of Infection in Field-Collected Bi. Snails

In Zimbabwe, neither cercarial shedding nor snail crushing detected any *S. mansoni* infection in field-collected snails. However, the smND1-LAMP assay identified 14 positive pooled *Bi. pfeifferi* samples among 68 tested pools, corresponding to a positive rate of 20.6% (14/68) (Table 3), indicating higher positive rate than microscopy. In addition, all five pooled *Bu. globosus* samples (the intermediate host of *S. haematobium*) tested negative by smND1-LAMP, with no observable colour change.

In Burkina Faso, microscopy similarly detected no positive cases among the 54 field-collected *Bi. pfeifferi* pooled snail samples. In contrast, the smND1-LAMP assay detected 14 positive pools (14/54, positive rate 25.9%), whereas conventional PCR detected 12 positive pools (12/54, positive rate 22.2%) (Table 4). Ten pools were positive by both smND1-LAMP and conventional PCR. Cohen’s kappa analysis showed substantial agreement (κ = 0.697) between smND1-LAMP and conventional PCR for field-collected *Bi. pfeifferi* pooled snails from Burkina Faso. To further confirm assay accuracy, four pooled samples that tested positive by both PCR and LAMP were subjected to sequencing. NCBI BLAST analysis showed that these sequences matched the reported mitochondrial ND1 gene fragment of *S. mansoni*.

## 4. Discussion

Molecular biology methods have been shown to detect *S. mansoni* infection in snails even when traditional parasitological techniques fail to identify positive cases [31]. In recent years, several LAMP-based assays targeting different genetic regions have been developed, demonstrating variable diagnostic performance [32]. The Sm1–7-based LAMP assay, targeting a 121 bp repetitive Sm1–7 sequence, has been widely applied and achieved 100% positive rates in human fecal samples, with sensitivities ranging from 78.3% to 87.4% [33,34]. Similarly, the SmMIT-LAMP assay, targeting a 620 bp mitochondrial minisatellite region, demonstrated 100% specificity and 85.7% sensitivity compared with PCR in both mouse feces and *Bi.* snails [35,36]. In contrast, the SmITS1-LAMP assay, targeting the 18S and 5.8S ribosomal internal transcribed spacer 1 (ITS1) region, showed limited sensitivity (17/144, 11.8%) for detecting *S. mansoni* infection in Kato–Katz-positive human fecal samples [37], likely due to its relatively higher detection limit [37]. Another study designed primers based on partial sequences of the 28S and 18S ITS regions for the *Biomphalaria*-LAMP assay to detect snail infections, highlighting that differences in genetic targets and methodological design can substantially influence diagnostic performance [38].

In this study, the smND1-LAMP assay was developed targeting a partial fragment of the mitochondrial ND1 gene of *S. mansoni*. The ND1 gene encodes a subunit of mitochondrial complex I [39]. Mitochondrial targets have high copy numbers, which is advantageous for sensitive detection, and comparative mitogenomic analysis is useful for identifying diagnostic markers in *Schistosoma* spp. [40]. However, mitochondrial genes may also show geographical variation among *S. mansoni* populations [41]. Such variation could potentially affect primer binding. Therefore, the selection of primer-binding regions that are genetically conserved within species but divergent among species remains important for reducing the risk of false negative results.

The optimized smND1-LAMP assay used four essential primers, including two inner primers and two outer primers, and was performed under isothermal conditions at 65 °C for 1 h, followed by enzyme inactivation at 80 °C for 10 min. Although loop primers can be added to accelerate LAMP, their inclusion may also increase the risk of non-specific amplification. In our preliminary test, rapid and stable positive amplification was achieved using only the four essential primers without loop primers. Therefore, the final smND1-LAMP assay used a four-primer system to maintain amplification efficiency while reducing the potential risk of false positive reactions. The assay demonstrated high specificity, with no cross-reactivity observed with other schistosome species or parasites, and capable detection limit of one copy of the *S. mansoni* pUC57/ND1 recombinant plasmid. The smND1-LAMP assay showed improved detection performance compared with microscopy and PCR, and similar performance comparable to SGPCR in this preliminary evaluation. Larger studies would be needed to formally establish non-inferiority between smND1-LAMP and SGPCR. Like other isothermal amplification methods [23], the smND1-LAMP assay requires only simple equipment and produces results within approximately 1 h, making it suitable for field and resource-limited settings. In laboratory-infected snails, it detected additional infections that were missed by microscopy, suggesting its ability to identify low-intensity infections. These findings are consistent with previous reports indicating that LAMP assays offer enhanced sensitivity for detecting schistosome infections [27,42,43].

During field surveys in Burkina Faso and Zimbabwe, no cercariae were observed by microscopy [30], likely due to low infection intensity or the presence of schistosomes at developmental stages other than the cercarial stage. In contrast, the smND1-LAMP assay successfully detected *S. mansoni* DNA in field-collected samples, highlighting its practical utility for field surveillance. As LAMP-based detection relies on DNA amplification rather than parasite morphology, it could theoretically detect infections across different developmental stages, providing a significant advantage over conventional parasitological methods.

The development of more sensitive diagnostic tools for detecting *S. mansoni* in intermediate host snails is therefore essential and needed for surveillance and control strategies. During field sampling in Zimbabwe, most water bodies were dry, and only five of the 14 schistosomiasis-endemic villages had accessible water sources. This observation suggests that establishing permanent snail monitoring sites, particularly during the dry season, could improve surveillance efficiency. Given the higher sensitivity of the smND1-LAMP assay, its application in such monitoring systems could facilitate early detection of transmission risk and support timely preventive interventions at the site of snail collection, thereby reducing human exposure to contaminated water.

As a country that previously experienced a high burden of schistosomiasis, China achieved transmission interruption of schistosomiasis in 2023 [44] and reached the WHO target for elimination as a public health problem by 2020, supported by long-term surveillance evidence [45]. LAMP-based detection has been used in China for the surveillance of *S. japonicum* infection in intermediate host snails during the elimination phase [46]. In the present study, we adapted this approach for *S. mansoni* by using a similar basic LAMP reaction system and closed-tube calcein-based visual detection, while redesigning the assay with *S. mansoni*-specific ND1 primers and optimizing the reaction conditions. We also refined the interpretation of LAMP results by combining visual colour change, agarose gel electrophoresis, and real-time turbidity measurement, which improved the reliability of result confirmation. Given the differences in parasite species, snail hosts, and transmission settings, direct transfer of diagnostic methods is not sufficient. Consequently, species-specific target selection and validation with relevant snail samples are required [47,48]. Our findings suggest that the smND1-LAMP assay may be a useful diagnostic tool for detecting *S. mansoni* infection in *Bi.* snails for both endemic and non-endemic settings, although larger field studies are still needed.

Several limitations should be acknowledged. First, the number of laboratory-infected snails was limited, which may affect the robustness of the sensitivity and specificity estimates. Therefore, the diagnostic performance figures should be interpreted as preliminary and may be benefit from validation in larger cohorts. Second, the absence of parasitologically confirmed positive snails in field-collected samples prevented a direct comparison between microscopy-based methods and the LAMP assay under field conditions. Third, early-stage infections were not included, and thus the ability of the assay to detect prepatent infections requires further validation. Fourth, although the primer-binding regions were selected from relatively conserved regions of the *S. mansoni* mitochondrial genome, validation using geographically diverse *S. mansoni* isolates is still needed to assess the potential impact of regional sequence polymorphisms on assay performance. Fifth, sequencing was performed only on selected LAMP-positive and PCR-positive samples whereas LAMP-positive and PCR-negative samples were not sequenced. Consequently, further investigation is required to elucidate the reasons for this discrepancy. In addition, environmental factors (e.g., water temperature, pH, and vegetation) and variations in snail populations (e.g., species composition and density) may influence detection efficiency.

Future studies should include larger and more diverse sample sets, particularly incorporating early-stage infections, to further validate assay performance. Comparison with established diagnostic PCR/SGPCR assays using independent primer sets would also be valuable for more comprehensive performance evaluation. The feasibility of pooled sample testing should also be explored to improve cost-effectiveness and operational efficiency. Furthermore, the development of portable, integrated LAMP platforms will be critical for facilitating on-site application and supporting routine surveillance and early warning systems in schistosomiasis-endemic regions.

## 5. Conclusions

A novel *S. mansoni*-specific smND1-LAMP assay was developed for the detection of *S. mansoni* infection in *Bi.* snails through primer screening, reaction optimization and performance evaluation. The smND1-LAMP assay targets a fragment of the mitochondrial ND1 gene and operates at 65 °C for 1 h, with a minimum detection limit of one copy of the pUC57/smND1 recombinant plasmid. The assay demonstrated high specificity, with no cross-reactivity observed against other parasites, including closely related schistosome species. In laboratory-infected snails, the smND1-LAMP assay outperformed microscopy and showed comparable performance to SGPCR, based on a preliminary study with limited samples. In field surveys conducted in Burkina Faso, smND1-LAMP yielded a higher positive rate than conventional PCR, whereas microscopy failed to detect any positive snails. These findings highlight the potential of smND1-LAMP as a sensitive, practical, and field-applicable tool for routine snail surveillance and early warning in schistosomiasis-endemic areas, thereby supporting improved disease monitoring and control strategies.

## Figures and Tables

**Table 1:** Sequences of all candidate LAMP primer sets.

Candidate Target	Primer	Sequence (5′ to 3′)
s-rRNA	s-rRNA-F3	TCGGTAATTTAAGACAGGTCTA
	s-rRNA-B3	AGCGCCTGATTCTTCAAG
	s-rRNA-FIP	CCTACTTCCGAGTCCTAAATCTGAGATGCTGCTAATATTATAGGTTGT
	s-rRNA-BIP	ATAGTTTACGTACACATCGCCCGTACCATGTTACGACTTAACTCA
ND1	ND1-F3	TTCAGTTGCGTAAGGGTC
	ND1-B3	CACCTATCGATACCACTTTGA
	ND1-FIP	ATTGAAACCATGCACTTTCACCTTTGGATTGGTACAGAGTTTTGC
	ND1-BIP	TCGAAGGTGATTTTCAGTATTTGGGAACATTCCGTAAATCTCAACC
CYTB	CYTB-F3	GAGAATATAAAGCCTGAGTGG
	CYTB-B3	AAGGATATTCAGCATGACAAG
	CYTB-FIP	GGCAACCATAAAACGAACAGAAAAATATATTATGCTATGCTTCGATCTG
	CYTB-BIP	ATTCTGTTTTTCGGCAGGTTAAATTCAATATACCCTAACCCTACACA
ND4	ND4-F3	TGCATATTGTCTCGAAGATGA
	ND4-B3	TCCTAACAATCTACCCCCA
	ND4-FIP	GCACCCATAAATACCATGACACTTATTTATCAACTGTATTTTGTTCGG
	ND4-BIP	AGAAGTGGGAGTGGCCGTTAAACAAAATCATGACTGCTGAT
COX3	COX3-F3	CGGTGCTTCCTTGCATAT
	COX3-B3	CGCTAGAACCCTTAATATACCAA
	COX3-FIP	CAAACTACATACTTGCGAAACAAGTATCATCCGTTTATTATGACAGTT
	COX3-BIP	TGTTACGGATTAGTTGTAAGATGGCGTAAGAAAAACAATTAACTCCCTAG

**Table 2:** Diagnostic performance of different methods for detecting *S. mansoni* infection in laboratory-maintained *Bi.* snails.

Detection Method	Infected Snails	Uninfected Snails	TP	TN	FP	FN	Sensitivity % (95% CI)	Specificity % (95% CI)
Cercarial shedding + microscopy	12	6	6	6	0	6	50 (21.1–78.9)	100 (54.1–100)
Snail crushing + microscopy	12	6	6	6	0	6	50 (21.1–78.9)	100 (54.1–100)
smND1-LAMP	12	6	9	6	0	3	75 (42.8–94.5)	100 (54.1–100)
SGPCR	12	6	9	6	0	3	75 (42.8–94.5)	100 (54.1–100)

TP = true positives; TN = true negatives; FP = false positives; FN = false negatives; CI = confidence interval.

**Table 3:** Detection of *S. mansoni* infection in field-collected *Bi. pfeifferi* snails from Zimbabwe.

Detection Method	Positive Snail Pools	Negative Snail Pools	Positive Rate
Cercarial shedding + microscopy	0	68	0.0%
Snail crushing + microscopy	0	68	0.0%
smND1-LAMP	14	54	20.6%

**Table 4:** Detection of *S. mansoni* infection in field-collected *Bi. pfeifferi* snails from Burkina Faso.

Detection Method	Positive Snail Pools	Negative Snail Pools	Positive Rate (%)
Cercarial shedding + microscopy	0	54	0.0
Conventional PCR	12	42	22.2
smND1-LAMP	14	40	25.9

## Data Availability

All relevant data, including primer sequences, are provided within the manuscript.

## Supplementary Materials

The following are available online at https://www.mdpi.com/article/10.3390/tropicalmed11060157/s1, Table S1: Parasite sample information used in this study; Table S2: Pair-wise sequence homology of the five candidate mitochondrial target regions between *S. mansoni* and representative *Schistosoma* species; Table S3: Candidate mitochondrial target regions screened for LAMP primer design.

## Abbreviations

The following abbreviations are used in this manuscript:

LAMP	Loop-mediated isothermal amplification
NTD	Neglected tropical disease
PCR	Polymerase chain reaction
SGPCR	SYBR green real-time PCR
ND1	NADH dehydrogenase subunit 1
WHO	World Health Organization
F3	Forward outer primer
FIP	Forward inner primer
BIP	Backward inner primer
B3	Backward outer primer
TP	True positives
TN	True negatives
FP	False positives
FN	False negatives
CI	Confidence interval

## References

[1] Farghaly A., Saleh A.A., Mahdy S., El-Khalik D.A., El-Aal N.F.A., Abdel-Rahman S.A., Salama M.A. (2016). Molecular approach for detecting early prepatent *Schistosoma mansoni* infection in *Biomphalaria alexandrina* snail host. J. Parasit. Dis..

[2] Bakuza J.S., Gillespie R., Nkwengulila G., Adam A., Kilbride E., Mable B.K. (2017). Assessing *S. mansoni* prevalence in *Biomphalaria snails* in the Gombe ecosystem of western Tanzania: The importance of DNA sequence data for clarifying species identification. Parasit. Vectors.

[3] Utzinger J., Raso G., Brooker S., De Savigny D., Tanner M., Ornbjerg N., Singer B.H., N’Goran E.K. (2009). Schistosomiasis and neglected tropical diseases: Towards integrated and sustainable control and a word of caution. Parasitology.

[4] Lackey E.K., Horrall S. (2023). Schistosomiasis. StatPearls.

[5] World Health Organization The Fact Sheets of Schistosomiasis (Bilharzia). https://www.who.int/schistosomiasis/en/.

[6] Chitsulo L., Engels D., Montresor A., Savioli L. (2000). The global status of schistosomiasis and its control. Acta Trop..

[7] Wang X.Y., Li Q., Li Y.L., Guo S.Y., Li S.Z., Zhou X.N., Guo J.G., Bergquist R., Juma S., Zhang J.F. (2024). Prevalence and correlations of schistosomiasis mansoni and schistosomiasis haematobium among humans and intermediate snail hosts: A systematic review and meta-analysis. Infect. Dis. Poverty.

[8] Dai S.M., Guan Z., Zhang L.J., Lv S., Cao C.L., Li S.Z., Xu J. (2020). Imported Schistosomiasis, China, 2010–2018. Emerg. Infect. Dis..

[9] Zhou X.N., Li S.Z., Xu J., Chen J.X., Wen L.Y., Zhang R.L., Lu C. (2019). Surveillance and control strategy of imported schistosomiasis mansoni: An expert consensus. Zhongguo Xue Xi Chong Bing Fang Zhi Za Zhi.

[10] Lv S., Xu J., Li Y.L., Bao Z.P., Zhang L.J., Yang K., Lin D.D., Liu J.B., Wang T.P., Ren G.H. (2025). Snail control as a crucial approach to schistosomiasis elimination: Evidence from the People’s Republic of China. Infect. Dis. Poverty.

[11] Zhang J.F., Wen L.Y., Xu J., Liang Y.S., Yan X.L., Ren G.H., Jia T.W., Wand W., Zhou X.N. (2019). Current status and transmission risks of oversea imported schistosomiasis in China. Chin. J. Schistosomiasis Control..

[12] World Health Organization (2020). Ending the Neglect to Attain the Sustainable Development Goals: A Road Map for Neglected Tropical Diseases 2021–2030.

[13] Ogongo P., Kariuki T.M., Wilson R.A. (2018). Diagnosis of schistosomiasis mansoni: An evaluation of existing methods and research towards single worm pair detection. Parasitology.

[14] World Health Organization (2022). WHO Guideline on Control and Elimination of Human Schistosomiasis.

[15] World Health Organization (2002). Prevention and Control of Schistosomiasis and Soiltransmitted Helminthiasis.

[16] Mutsaka-Makuvaza M.J., Zhou X.N., Tshuma C., Abe E., Manasa J., Manyangadze T., Allan F., Chin’ombe N., Webster B., Midzi N. (2020). Genetic diversity of *Biomphalaria pfeifferi*, the intermediate host of *Schistosoma mansoni* in Shamva district, Zimbabwe: Role on intestinal schistosomiasis transmission. Mol. Biol. Rep..

[17] Colley D.G., King C.H., Kittur N., Ramzy R.M.R., Secor W.E., Fredericks-James M., Ortu G., Clements M.N., Ruberanziza E., Umulisa I. (2020). Evaluation, Validation, and Recognition of the Point-of-Care Circulating Cathodic Antigen, Urine-Based Assay for Mapping *Schistosoma mansoni* Infections. Am. J. Trop. Med. Hyg..

[18] Garcia-Bernalt Diego J., Fernandez-Soto P., Febrer-Sendra B., Crego-Vicente B., Muro A. (2021). Loop-Mediated Isothermal Amplification in Schistosomiasis. J. Clin. Med..

[19] Frickmann H., Hahn A., Eberhardt K.A., Loderstadt U., Schwarz N.G., Hagen R.M. (2026). Matrix-Dependent Sensitivity of Two Pan-Trematode PCR Assays for Detecting *Schistosoma* spp. in Clinical Human Samples. Infect. Dis. Rep..

[20] Weerakoon K.G., Gordon C.A., McManus D.P. (2018). DNA Diagnostics for Schistosomiasis Control. Trop. Med. Infect. Dis..

[21] Qin Z.Q., Xu J., Feng T., Lv S., Qian Y.J., Zhang L.J., Li Y.L., Lv C., Bergquist R., Li S.Z. (2018). Field Evaluation of a Loop-Mediated Isothermal Amplification (LAMP) Platform for the Detection of *Schistosoma japonicum* Infection in *Oncomelania hupensis* Snails. Trop. Med. Infect. Dis..

[22] Deng W., Wang S., Wang L., Lv C., Li Y., Feng T., Qin Z., Xu J. (2022). Laboratory Evaluation of a Basic Recombinase Polymerase Amplification (RPA) Assay for Early Detection of *Schistosoma japonicum*. Pathogens.

[23] Mesquita S.G., Gadd G., Coelho F.S., Cieplinski A., Emery A., Lugli E.B., Simoes T.C., Fonseca C.T., Caldeira R.L., Webster B. (2024). Laboratory and field validation of the recombinase polymerase amplification assay targeting the *Schistosoma mansoni* mitochondrial minisatellite region (SmMIT-RPA) for snail xenomonitoring for schistosomiasis. Int. J. Parasitol..

[24] Notomi T., Okayama H., Masubuchi H., Yonekawa T., Watanabe K., Amino N., Hase T. (2000). Loop-mediated isothermal amplification of DNA. Nucleic Acids Res..

[25] Li Y., Guo S., Dang H., Zhang L., Xu J., Li S. (2023). *Oncomelania hupensis* Distribution and Schistosomiasis Transmission Risk in Different Environments under Field Conditions. Trop. Med. Infect. Dis..

[26] Li Y.L., Dang H., Guo S.Y., Zhang L.J., Feng Y., Ding S.J., Shan X.W., Li G.P., Yuan M., Xu J. (2022). Molecular evidence on the presence of *Schistosoma japonicum* infection in snails along the Yangtze River, 2015–2019. Infect. Dis. Poverty.

[27] Li H.M., Qin Z.Q., Bergquist R., Qian M.B., Xia S., Lv S., Xiao N., Utzinger J., Zhou X.N. (2021). Nucleic acid amplification techniques for the detection of *Schistosoma mansoni* infection in humans and the intermediate snail host: A structured review and meta-analysis of diagnostic accuracy. Int. J. Infect. Dis..

[28] Vaillant M.T., Philippy F., Neven A., Barre J., Bulaev D., Olliaro P.L., Utzinger J., Keiser J., Garba A.T. (2024). Diagnostic tests for human Schistosoma mansoni and *Schistosoma haematobium* infection: A systematic review and meta-analysis. Lancet Microbe.

[29] Mutsaka-Makuvaza M.J., Zhou X.N., Tshuma C., Abe E., Manasa J., Manyangadze T., Allan F., Chinómbe N., Webster B., Midzi N. (2020). Molecular diversity of *Bulinus* species in Madziwa area, Shamva district in Zimbabwe: Implications for urogenital schistosomiasis transmission. Parasit. Vectors.

[30] Midzi N., Mutsaka-Makuvaza M.J., Lv S., Qiang Q.Z., Li H.M., Tang L., Yu X.L., Li C.L., Manengureni T., Soko W. (2025). Village and age based precision mapping of schistosomiasis and soil-transmitted helminths in Chevakadzi ward of Shamva district in Zimbabwe. Sci. Rep..

[31] Gandasegui J., Fernández-Soto P., Muro A., Simões Barbosa C., Lopes de Melo F., Loyo R., de Souza Gomes E.C. (2018). A field survey using LAMP assay for detection of *Schistosoma mansoni* in a low-transmission area of schistosomiasis in Umbuzeiro, Brazil: Assessment in human and snail samples. PLoS Negl. Trop. Dis..

[32] Zhou X., Li J., Qiu J., Feng T., Lv C., Deng W., Bergquist R., Xu J., Li S., Qin Z. (2025). Test accuracy of loop-mediated isothermal amplification for schistosomiasis in low endemicity areas: A systematic review and meta-analysis. Infect. Dis. Poverty.

[33] Allam A.F., Kamel M.A., Farag H.F., Raheem H.G., Shehab A.Y., Hagras N.A. (2022). Performance of loop-mediated isothermal amplification (LAMP) for detection of *Schistosoma mansoni* infection compared with Kato-Katz and real-time PCR. J. Helminthol..

[34] Price M., Cyrs A., Sikasunge C.S., Mwansa J., Lodh N. (2019). Testing the Infection Prevalence of *Schistosoma mansoni* after Mass Drug Administration by Comparing Sensitivity and Specificity of Species-Specific Repeat Fragment Amplification by PCR and Loop-Mediated Isothermal Amplification. Am. J. Trop. Med. Hyg..

[35] Mesquita S.G., Neves F., Scholte R.G.C., Carvalho O.D.S., Fonseca C.T., Caldeira R.L. (2021). A loop-mediated isothermal amplification assay for *Schistosoma mansoni* detection in *Biomphalaria* spp. from schistosomiasis-endemic areas in Minas Gerais, Brazil. Parasit. Vectors.

[36] Fernández-Soto P., Gandasegui Arahuetes J., Sánchez Hernández A., López Abán J., Vicente Santiago B., Muro A. (2014). A loop-mediated isothermal amplification (LAMP) assay for early detection of *Schistosoma mansoni* in stool samples: A diagnostic approach in a murine model. PLoS Negl. Trop. Dis..

[37] Gomes E.C.S., Barbosa Júnior W.L., Melo F.L. (2022). Evaluation of SmITS1-LAMP performance to diagnosis schistosomiasis in human stool samples from an endemic area in Brazil. Exp. Parasitol..

[38] Gandasegui J., Fernández-Soto P., Hernández-Goenaga J., López-Abán J., Vicente B., Muro A. (2016). *Biompha*-LAMP: A New Rapid Loop-Mediated Isothermal Amplification Assay for Detecting *Schistosoma mansoni* in *Biomphalaria glabrata* Snail Host. PLoS Negl. Trop. Dis..

[39] National Center for Biotechnology Information (2021). ND1 NADH Dehydrogenase Subunit 1 [*Schistosoma mansoni*]. https://www.ncbi.nlm.nih.gov/gene/800022.

[40] Zarowiecki M.Z., Huyse T., Littlewood D.T.J. (2007). Making the most of mitochondrial genomes—Markers for phylogeny, molecular ecology and barcodes in *Schistosoma* (Platyhelminthes: Digenea). Int. J. Parasitol..

[41] Morgan J.A., Dejong R.J., Adeoye G.O., Ansa E.D., Barbosa C.S., Bremond P., Cesari I.M., Charbonnel N., Correa L.R., Coulibaly G. (2005). Origin and diversification of the human parasite *Schistosoma mansoni*. Mol. Ecol..

[42] Ally O., Kanoi B.N., Ochola L., Nyanjom S.G., Shiluli C., Misinzo G., Gitaka J. (2024). Schistosomiasis diagnosis: Challenges and opportunities for elimination. PLoS Negl. Trop. Dis..

[43] Salas-Coronas J., Luzón-García M.P., Crego-Vicente B., Soriano-Pérez M.J., Febrer-Sendra B., Vázquez-Villegas J., Diego J.G., Cabeza-Barrera I.M., Castillo-Fernández N., Muro A. (2023). Evaluation of Loop-Mediated Isothermal Amplification (LAMP) in Urine Samples for the Diagnosis of Imported Schistosomiasis. Trop. Med. Infect. Dis..

[44] Zhang L., He J., Yang F., Dang H., Li Y., Guo S., Li S., Cao C., Xu J., Li S. (2024). Progress of schistosomiasis control in People’s Republic of China in 2023. Zhongguo Xue Xi Chong Bing Fang Zhi Za Zhi.

[45] Xu J., Li S.Z., Zhang L.J., Bergquist R., Dang H., Wang Q., Lv S., Wang T.P., Lin D.D., Liu J.B. (2020). Surveillance-based evidence: Elimination of schistosomiasis as a public health problem in the Peoples’ Republic of China. Infect. Dis. Poverty.

[46] Guo S., Zhang L., Li Y., Zhang S., Xu X., Li Y., Cao C., Xu J., Li S. (2025). One Health integrated surveillance: A way forward to accelerate schistosomiasis elimination in China. Sci. One Health.

[47] Lv S., Xu J., Cao C.L., Zhang L.J., Li S.Z., Zhou X.N. (2019). China fighting against schistosomiasis for 70 years: Progress and experience. Zhongguo Xue Xi Chong Bing Fang Zhi Za Zhi.

[48] Li H.M., Arthur Djibougou D., Lu S.N., Lv S., Zongo D., Wang D.Q., Ding W., Qian Y.J., Huang L.L., Guan Y.Y. (2022). Strengthening capacity-building in malaria and schistosomiasis control under China-Africa cooperation: Assessing a case study of Burkina Faso. Sci. One Health.

