# Supplementary material for: Rapid and Sensitive Detection of Schistosoma mansoni in the Intermediate Snail Hosts Using Loop-Mediated Isothermal Amplification (LAMP) Diagnostics
Source: Trop Med Infect Dis. 2026 Jun 11;11(6):157. doi: 10.3390/tropicalmed11060157 (PMC13307744; doi:10.3390/tropicalmed11060157)
Supplement: Supplementary file 1 [file tropicalmed-11-00157-s001.zip › tropicalmed-4278572-supplementary.pdf]

Article

Rapid and sensitive detection of Schistosoma mansoni in the intermediate snail hosts using loop-mediated isothermal amplification (LAMP) diagnostics

Supplementary Materials

Table S1 Parasite sample information used in this study

Parasite species	Life cycle stage	Geographic origin	Sample source
S. mansoni	Adult worm	NIPD laboratory strain	NIPD repository
S. mansoni	Cercariae	NIPD laboratory strain	Extracted DNA
S. japonicum	Adult worm	Hubei Province, China	NIPD repository
S. haematobium	Eggs	Zimbabwe*	Extracted DNA
C. sinensis	Adult worm	Guangxi Province, China	NIPD repository
P. westermani	Adult worm	Yunnan Province, China	NIPD repository
E. granulosus	Adult worm	Sichuan Province, China	NIPD repository

*Provided by collaborators in Zimbabwe

Table S2. Pair-wise sequence homology of the five candidate mitochondrial target regions between *S. mansoni* and representative *Schistosoma* species

Source	s-rRNA homology (%)	ND1 homology (%)	CYTB homology (%)	ND4 homology (%)	COX3 homology (%)
S. mansoni	100.0	100.0	100.0	100.0	100.0
S. haematobium	82.1	66.4	66.4	67.3	64.5
S. japonicum	74.4	65.0	65.0	63.9	60.2
S. mekongi	71.0	62.1	62.1	65.9	63.6

Pair-wise sequence homology was calculated by comparing each candidate mitochondrial target region of *S. mansoni* with the corresponding regions of representative *Schistosoma* species. Sequence divergence can be calculated as 100% minus pair-wise homology.

Table S3. Candidate mitochondrial target regions screened for LAMP primer design

Candidate target	Description	GenBank accession	Reference
s-rRNA	Small ribosomal RNA	NC_002545	https://www.ncbi.nlm.nih.gov/nuccore/NC_002545.1?from=3686&to=4437
ND1	NADH dehydrogenase subunit 1	NC_002545	https://www.ncbi.nlm.nih.gov/gene/800022
CYTB	Cytochrome b	NC_002545	https://www.ncbi.nlm.nih.gov/gene/800017
ND4	NADH dehydrogenase subunit 4	NC_002545	https://www.ncbi.nlm.nih.gov/gene/800014
COX3	Cytochrome c oxidase subunit III	NC_002545	https://www.ncbi.nlm.nih.gov/gene/800016

NADH = nicotinamide adenine dinucleotide hydrogen.
